# Inhibition of Mammalian Target of Rapamycin (mTOR) Signaling in the Insular Cortex Alleviates Neuropathic Pain after Peripheral Nerve Injury

**DOI:** 10.3389/fnmol.2017.00079

**Published:** 2017-03-21

**Authors:** Minjee Kwon, Jeongsoo Han, Un Jeng Kim, Myeounghoon Cha, Sun Woo Um, Sun Joon Bai, Seong-Karp Hong, Bae Hwan Lee

**Affiliations:** ^1^Department of Physiology, Yonsei University College of MedicineSeoul, South Korea; ^2^Brain Korea 21 PLUS Project for Medical Science, Yonsei University College of MedicineSeoul, South Korea; ^3^Department of Anesthesiology and Pain Medicine, Yonsei University College of MedicineSeoul, South Korea; ^4^Division of Bio and Health Sciences, Mokwon UniversityDaejeon, South Korea; ^5^Brain Research Institute and Epilepsy Research Institute, Yonsei University College of MedicineSeoul, South Korea

**Keywords:** neuropathic pain, mTOR, insular cortex, rapamycin, synaptic plasticity

## Abstract

Injury of peripheral nerves can trigger neuropathic pain, producing allodynia and hyperalgesia via peripheral and central sensitization. Recent studies have focused on the role of the insular cortex (IC) in neuropathic pain. Because the IC is thought to store pain-related memories, translational regulation in this structure may reveal novel targets for controlling chronic pain. Signaling via mammalian target of rapamycin (mTOR), which is known to control mRNA translation and influence synaptic plasticity, has been studied at the spinal level in neuropathic pain, but its role in the IC under these conditions remains elusive. Therefore, this study was conducted to determine the role of mTOR signaling in neuropathic pain and to assess the potential therapeutic effects of rapamycin, an inhibitor of mTORC1, in the IC of rats with neuropathic pain. Mechanical allodynia was assessed in adult male Sprague-Dawley rats after neuropathic surgery and following microinjections of rapamycin into the IC on postoperative days (PODs) 3 and 7. Optical recording was conducted to observe the neural responses of the IC to peripheral stimulation. Rapamycin reduced mechanical allodynia and downregulated the expression of postsynaptic density protein 95 (PSD95), decreased neural excitability in the IC, thereby inhibiting neuropathic pain-induced synaptic plasticity. These findings suggest that mTOR signaling in the IC may be a critical molecular mechanism modulating neuropathic pain.

## Introduction

Neuropathic pain is defined as pain initiated or caused by a primary lesion or dysfunction in the nervous system (Merskey and Bogduk, 1994). It is characterized by allodynia, hyperalgesia and spontaneous pain (Jaggi and Singh, 2011). Advancements in the understanding of chronic pain followed the suggestion of a “pain memory” by Melzack (1990). Since then, studies have indicated that neuropathic pain is intimately related to structural changes in the central nervous system (CNS), known as neuronal plasticity (Banic et al., 2004; May, 2008; Géranton et al., 2009; Latremoliere and Woolf, 2009; Seminowicz et al., 2009; Saab, 2012; Blom et al., 2014).

Plasticity of neuronal synapses requires protein synthesis and regulation for the formation of long-term memory (Banko et al., 2005). Mammalian target of rapamycin (mTOR), a serine-threonine protein kinase, initiates mRNA translation plays in the nervous system and plays important roles in cell proliferation and differentiation (Huang and Houghton, 2003; Liang et al., 2013). The activation of mTOR complex 1 (mTORC1) regulates protein synthesis by phosphorylating downstream effectors, such as eukaryotic initiation factor 4E binding protein 1 (4EBP1) and p70 ribosomal S6 protein kinase (p70S6K), which influence a wide range of physiological and pathological states (Hay and Sonenberg, 2004; Jaworski and Sheng, 2006; Swiech et al., 2008), including neuropathic, inflammatory and cancer-related pain (Jiménez-Díaz et al., 2008; Géranton et al., 2009; Norsted Gregory et al., 2010). One study showed that mTOR signaling regulates pain-related synaptic plasticity in the entorhinal-hippocampal pathway in a model of persistent peripheral nociception induced by peripheral bee venom injections (Lyu et al., 2013). Moreover, the levels of mTOR and extracellular signal-regulated kinase (ERK)1 and ERK2 were increased in the cerebrospinal fluid-contacting nucleus of the brainstem after nerve injury (Li et al., 2015). In the spinal cords of rats with chronic constriction injury, inhibition of mTOR with rapamycin reduces the expression of postsynaptic density protein 95 (PSD95; Zhang et al., 2013). PSD95 is enriched at excitatory synapses and mediates important interactions with receptors, such as Nmethyl-D-aspartate (NMDA) and a-amino-3-hydroxy-5-methyl-4-isoxazolepropionic acid (AMPA) receptors (Kim et al., 2007), which are essential elements in pain hypersensitivity (Toyoda et al., 2009). Thus, PSD95 may have a role in synaptic plasticity and pain hypersensitivity (Kalia et al., 2008; Kessels and Malinow, 2009; Yasaka et al., 2009; Liu and Salter, 2010; Zhou et al., 2011).

Clinical and basic studies persistently indicate critical roles of the insular cortex (IC) in pain processing (Burkey et al., 1999; Coffeen et al., 2011; Liu et al., 2013). Zhuo (2016) reported plastic changes and long-term potentiation (LTP) in the IC after injury. Moreover, human fMRI studies document a relationship between the IC and chronic neuropathic pain conditions (Garcia-Larrea and Peyron, 2013; Tseng et al., 2013), and interestingly, rostral regions of the IC are activated during noxious somatosensory stimulation (Coghill et al., 1999; Brooks et al., 2002). The rostral agranular IC (RAIC) in rats is essential for modulating nociception (Wu et al., 2016) and is implicated in pain behavior (Burkey et al., 1996; Jasmin et al., 2003). Recently, Jung et al. (2016) found that lesions in RAIC diminished pain-related behaviors in neuropathic pain models. These results suggest that the rostral part of the IC plays a crucial role in pain processing.

Therefore, the aim of this study was to investigate the role of the mTORC1 pathway in the IC after nerve injury. In particular, we focused on the relationship between the mTORC1 signaling and PSD95 or NMDA receptor 2B (NMDAR2B), which has been implicated to be associated with synaptic plasticity. The findings from our research may provide the foundation for a new molecular target, the mTOR pathway, for understanding neuropathic pain and may contribute to potential therapeutic applications.

## Materials and Methods

### Experimental Animals

Adult male Sprague-Dawley rats, weighing 250–280 g (Harlan, Koatec, Pyeongtaek, Korea) were housed in plastic cages and allowed to acclimate to the colony room for 7 days after arrival. Rats were maintained under 12-h light/dark cycles with pellets and water provided *ad libitum*. All experimental protocols in this study were in compliance with the National Institutes of Health guidelines and approved by the Institutional Animal Care and Use Committee of Yonsei University Health System (permit no: 2013-0276).

### Cannula Implantation

Rats were anesthetized with sodium pentobarbital (50 mg/kg, intraperitoneal ,[i.p.]) and in a stereotaxic frame. Guide cannulae (28-gauge) were bilaterally implanted into the RAIC (1.0 mm anterior to bregma, ±4.7 mm lateral from the midline, 5.8 mm beneath the surface of the skull; Han et al., 2015; Jung et al., 2016; Wu et al., 2016). Rats were allowed to recover for 1 week after cannula implantation.

### Neuropathic Surgery

Under sodium pentobarbital (50 mg/kg, i.p.) anesthesia, branches of the left sciatic nerve were exposed. The tibial and sural nerves were tightly ligated with 4-0 black silk and cut, whereas the common peroneal nerve was left intact (nerve-injured, NP group; Lee et al., 2000). Animals in the sham group underwent the same procedure for exposing the sciatic nerve but without injury.

### Mechanical Allodynia Test

Mechanical hypersensitivity of the hind paw was assessed before nerve injury and on postoperative day (POD) 1, 3 and 7 by a researcher blinded to the experimental conditions. Rats were habituated for 30 min to the test chambers, a metal mesh floor under plastic domes. Mechanical allodynia was measured by assessing thresholds for hind paw withdrawal to stimulation by an electrical von Frey filament (UGO Basile, Varese, Italy). Licking or rapid withdrawal of the hind paw was considered as a positive response. The responses were measured eight times in 2–3 min intervals. The mechanical forces were recorded for each withdrawal of the hind paw. Testing was also performed before and at 0.5, 1, 2, 4, 8, 12, 24 and 48 h after rapamycin microinjection.

### Rapamycin Microinjection into the IC

Rapamycin (R-5000; LC Laboratories, Woburn, MA, USA) was prepared in 0.06% dimethyl-sulfoxide (DMSO) diluted in saline. Microinjections were performed on POD3 and POD7. Rapamycin (0.5 μl of 600 nM) or an equivalent amount of vehicle solution was infused into the IC bilaterally through the injection cannulae using Hamilton syringes and PE-10 tubing. The injection cannula was maintained in position for at least 1 min to allow for drug absorption.

### Immunohistochemical Staining

Rats were anesthetized with urethane (1.25 g/kg, i.p.) and perfused transcardially with normal saline (0.9% NaCl) followed by a 4% solution of formaldehyde in 0.1 M sodium phosphate buffer (pH 6.8). The brains were extracted and post-fixed overnight at 4°C before cryoprotecting in 30% sucrose in phosphate-buffered saline (PBS, pH 7.4) for 24 h. Coronal tissue sections (30 μm) were cut using a cryostat (Microm HM525; Thermo Scientific, Waltham, MA, USA). The sections were incubated with PBS containing 1% normal horse serum for 30 min, and then incubated overnight in anti-c-Fos (AB 1584, 1:4000; Merck Millipore, Darmstadt, Germany) and phospho (p)-ERK (no. 5683, 1:2000; Cell Signaling Technology, Danvers, MA, USA) antibodies at 4°C. The sections were washed with PBS and incubated for 30 min in biotinylated antibody (1:50, Vector Laboratories, Burlingame, CA, USA). The slides were then washed again and incubated for 30 min with PBS containing the avidin-biotinylated horseradish peroxidase complex (ABC kit; Vector Laboratories). After washing, sections were incubated for 5–7 min in a PBS solution containing 0.1% 3,3′-diaminobenzidine tetrahydrochloride (DAB), 0.1% ammonium nickel sulfate, and 10 μl of H_2_O_2_. The reaction was terminated by washing with PBS. The slides were then dehydrated with ethanol, cleared in xylene and covered with Permount (Thermo Scientific).

c-Fos- and p-ERK-labeled cells in the ipsilateral and contralateral sites of the rostral IC defined by the rat brain atlas (Paxinos and Watson, 2006) were observed under light-field microscopy (20× objective; Olympus, Tokyo, Japan). To quantify c-Fos- and p-ERK-positive cells in the IC, a maximum of eight representative sections of the IC were chosen at random from each rat and counted by an observer blinded to the experimental conditions. The numbers of positive cells from each section were averaged to represent the cell count of each animal. Regions of interest (ROIs) were set for the ipsilateral and contralateral sides.

### Western Blot Analysis

On POD 3 and 7, rats received microinjections of vehicle solution or rapamycin as described above and were anesthetized with Enflurane for decapitation and IC tissue sample collection. The ipsilateral and contralateral rostral ICs were quickly dissected, frozen on dry ice, and stored at −70°C. For protein extraction, samples were homogenized in lysis buffer (ProPrep; Intron Biotechnology, Pyeongtaek, Korea) containing phosphatase inhibitors (Phosstop; Roche, Mannheim, Germany). Samples were then centrifuged at 12,000× g for 20 min at 4°C, and the supernatants were collected. Total protein concentrations were assessed with a spectrophotometer (Nano Drop ND-1000; Thermo Scientific), and 30 μg of protein per well were denatured and run on 10% gels (Bio-Rad, Hercules, CA, USA). Proteins were transferred onto a polyvinylidene difluoride membrane (Merck Millipore, Darmstadt, Germany), and the membranes were blocked by incubating in 3% skim milk. Membranes were incubated with primary antibodies against mTOR (no. 2972, 1:1000; Cell Signaling Technology), p-mTOR (Ser 2448, no. 5536, 1:500; Cell Signaling Technology), P70S6K (no. 9202, 1:1000; Cell Signaling Technology), p-P70S6K (Thr 389, no. 9234, 1:500; Cell Signaling Technology), 4EBP (no. 9452, 1:1000; Cell Signaling Technology), p-4EBP (Thr37/46, no. 2855, 1:500; Cell Signaling Technology), PSD95 (no. 3450, 1:1000; Cell Signaling Technology), NMDAR2B (no. 4207, 1:1000; Cell Signaling Technology) and β-actin (no. 3700, 1:10,000; Cell Signaling Technology). Membranes were then incubated with the appropriate anti-rabbit or anti-mouse horseradish peroxidase-conjugated secondary antibodies (no. 7074 and 7076, 1:10,000; Cell Signaling Technology). Proteins were visualized by applying a chemiluminescent substrate (GE Healthcare, Little Chalfont, UK) and observed using the LAS system (LAS 4000; GE Healthcare). The signals for phosphorylated proteins were normalized to those from the nonspecific forms. β-actin was used as the loading control.

### Optical Imaging

Optical imaging was conducted as described in a previous article (Han et al., 2016) with a slight modification. Before imaging, 16 Sprague-Dawley male rats (250–300 g; *n* = 8/group) were deeply anesthetized with urethane (1.25 g/kg, i.p.) on POD 7 and given atropine (5 mg/kg, i.p.) to suppress mucus secretion and dexamethasone sulfate (1 mg/kg, i.p.) to reduce swelling of the brain. Rats were then placed on their sides in a custom-made stereotaxic frame, which exposed the IC located in the anterolateral aspect of the brain, and endotracheally intubated to minimize respiratory movements during optical imaging. Heart rates were monitored by electrocardiography and body temperatures were maintained at 36°C with a rectal probe and heating pad system (homeothermic blanket control unit; Harvard Apparatus, Holliston, MA, USA). Lidocaine was applied to the right temporal muscle and the skin contralateral to the injury. The skin overlying the temporalis muscle and zygomatic arch was carefully removed to prevent excessive bleeding. Craniectomies were performed and the overlying dura were resected to expose the cortex. The cortices were stained using a voltage-sensitive dye (di-2-ANEPEQ, 50 μg/mL in saline; Molecular Probes, Eugene, OR, USA) for 1 h and carefully rinsed with saline. Imaging was performed before and after the application of vehicle solution or 600 nM rapamycin directly to the exposed cortex for 30 min. The changes in fluorescence were measured using a high-resolution CCD camera (Brainvision, Inc., Tokyo, Japan) equipped with a dichroic mirror with a 510–550 nm excitation filter and 590 nm absorption filter. A tungsten halogen lamp (150 W) was used for excitation of fluorescence. The imaging area was 6.4 × 4.8 mm^2^ and consisted of 184 × 124 pixels.

A pair of stainless steel electrodes was inserted into the left hind paws where the electronic von Frey filaments had been applied during behavioral testing. The stimulation was a square pulse (width: 0.1 ms, interstimulus interval: 5 s, intensity: 5.0 mA) using a stimulus isolation unit (World Precision Instruments, Sarasota, FL, USA). The fluorescence intensity during each trial was detected for approximately 940 ms under an optical microscope (Leica Microsystems, Ltd., Heerbrugg, Switzerland) equipped with a 1× objective and a 1× projection lens. Optical signals were acquired at a rate of 3.7 ms/frame and averages of 30 trials were recorded by an optical imaging recording system (MiCAM02; Brainvision, Inc.). Optical imaging acquisition was triggered by electrocardiogram signals using a stimulus/non-stimulus subtraction method. After optical imaging, rats were euthanized by an overdose of urethane.

To normalize the value of each pixel, the ratio of the intensity of fluorescence (ΔF) in each pixel relative to the initial fluorescence intensity (F) was expressed as the fractional change (ΔF/F). Amplitudes and excitatory areas of optical signals were measured using a spatial filter (9 × 9 pixels) to reduce artifacts caused by vibration and brain movements. Using captured images, fractional changes in optical signals (optical intensity) and areas of activation were quantified. Changes in optical intensity in the IC were expressed as a percentage of fractional change in fluorescence (ΔF/F × 100). Activated areas were analyzed using the activated pixel number of an ROI (previously described in Han et al., 2016)/total pixel number of the ROI × 100. Data were collected and analyzed with BV Analyzer software (Brainvision, Inc.).

### Statistical Analysis

Data from the behavioral tests were analyzed by two-way analysis of variance (ANOVAs) with repeated measures, with group and POD as factors, and by unpaired *t* tests for *post hoc* comparisons between groups. Unpaired *t* tests were performed for the analyses of immunohistochemistry, Western blotting and optical imaging data. Statistical analyses were performed by SPSS 20.0 software (IBM Corporation, Armonk, NY, USA). All values are expressed as means ± SEM. *P* values less than 0.05 were considered statistically significant.

## Results

### Nerve Injury Leads to the Development of Mechanical Allodynia

**Figure 1:**
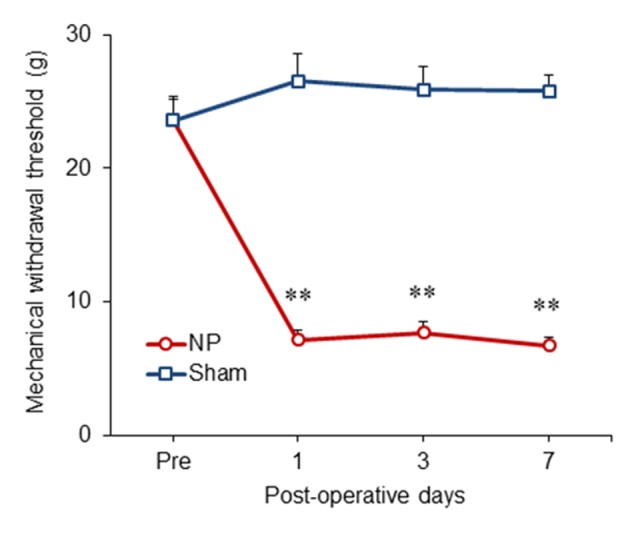
**Development of mechanical allodynia in nerve-injured (NP) and sham-injured (Sham) rats.** After nerve injury, rats developed significant neuropathic pain on postoperative day 1 (POD1), POD3 and POD7 compared with the sham group. Data are presented as means ± SEM. **P < 0.01 vs. Sham.

We examined the time course of behavioral changes in our neuropathic pain model (Lee et al., 2000) using the thresholds of the injured hind paws at 1, 3 and 7 days after the neuropathic pain surgery. From POD1 to POD7, the thresholds between NP and sham groups (*n* = 6) were significantly different (group, *F*_(1,10)_ = 118.917, *P* < 0.001; POD, *F*_(3,30)_ = 16.159, *P* < 0.001; group and POD interaction, *F*_(3,30)_ = 29.537, *P* < 0.001; two-way repeated measured ANOVA; Figure 1). The mechanical thresholds of the peripheral nerve injury group were decreased compared with those of the shams on POD1 (unpaired *t* test, *P* < 0.001), POD3 (unpaired *t* test, *P* < 0.001) and POD7 (unpaired *t* test, *P* < 0.001).

### Increased c-Fos and p-ERK Levels in the IC of Rats after Nerve Injury

**Figure 2:**
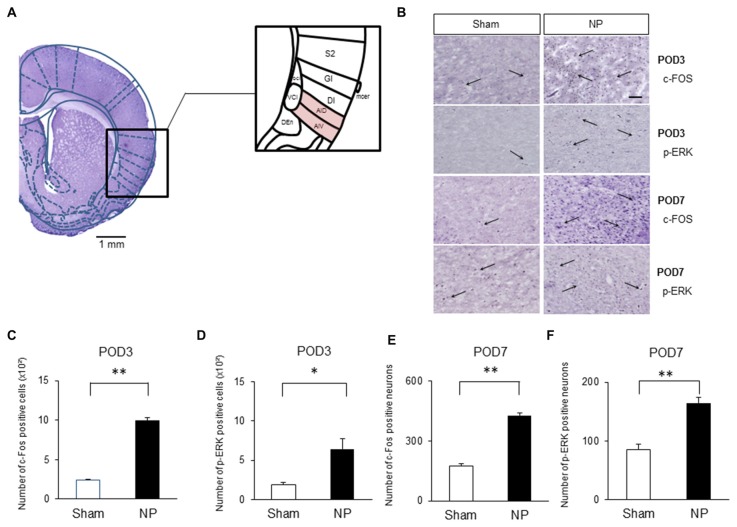
**c-Fos and phospho (p)-extracellular signal-regulated kinase (ERK) expression in NP and Sham rats. (A)** Histological clarification of rostral insular cortex (IC) with rat atlas. Subdivisions of the IC were included in the black square box. c-Fos- and p-ERK-positive cells in the AIV and AID were analyzed. AIV, agranular insular cortex ventral; AID, agranular insular cortex dorsal; DI, dysgranular insular cortex; GI, granular insular cortex; S2, secondary somatosensory cortex; Den, dorsal endopiriform nucleus; VCI, ventral part of claustrum; DCI, dorsal part of claustrum. **(B)** Microphotographs of c-FOS and p-ERK in the rostral IC (AIV and AID areas). The arrows mean positive cells for c-FOS or p-ERK. **(C)** Quantification of c-Fos- and **(D)** p-ERK-positive cells on POD3. **(E)** Quantification of c-Fos- and **(F)** p-ERK-positive cells on POD7. Scale bars, 200 μm. Data are presented as means ± SEM. *P < 0.05, **P < 0.01 vs. Sham.

Several authors have proposed that the IC is activated under various type of pain, including neuropathic pain (Alvarez et al., 2009; Qiu et al., 2013). Figure 2A shows histological confirmation of rostral IC with Paxinos and Watson’s rat atlas (Paxinos and Watson, 2006). To confirm that the rostral IC is involved with neuropathic pain, immunohistochemistry was performed to assess the numbers of cells expressing c-Fos and p-ERK, which are important markers for nociceptive neuronal activation in the CNS (Gao and Ji, 2009). On POD3, rats in the NP group had higher numbers of c-Fos- (Figure 2B, the first row microphotographs and Figure 2C, the graph of c-Fos-positive neurons, NP group (*n* = 5), Sham group (*n* = 5), *P* < 0.001, unpaired *t* test) and p-ERK- (Figure 2B, the second row microphotographs and Figure 2D, the graph of p-ERK positive cells, NP group (*n* = 4), Sham group (*n* = 4), *P* < 0.05, unpaired *t* test, scale bar: 200 μm) positive cells than sham animals. The numbers of cells positive for c-Fos (Figure 2B, the third row microphotographs and Figure 2E, the graph of c-Fos-positive cells, NP group (*n* = 6), Sham group (*n* = 6), *P* < 0.01, unpaired *t* test) and p-ERK (Figure 2B, the fourth row microphotographs and Figure 2F, the graph of p-ERK-positive neurons, NP group (*n* = 6), Sham group (*n* = 6), *P* < 0.01, unpaired *t* test) in nerve-injured rats were also significantly higher than in sham controls on POD7.

### Nerve Injury Activates mTOR Signaling, PSD95 and NMDAR2B Expressions in the IC

**Figure 3:**
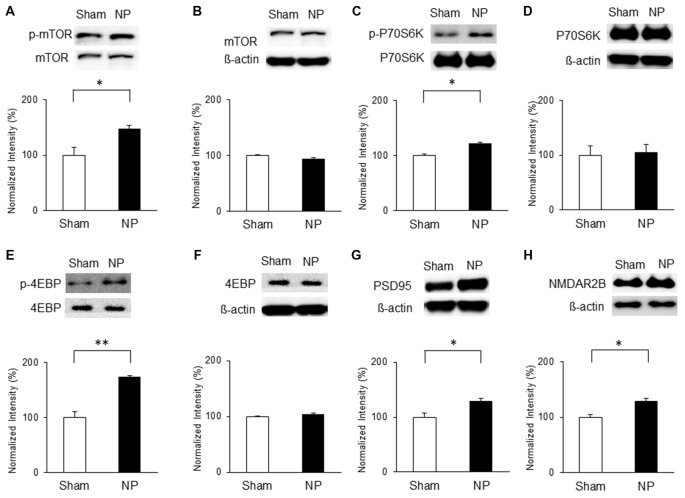
**Phosphorylation of mammalian target of rapamycin (mTOR), p70 ribosomal S6 protein kinase (P70S6K) and 4E binding protein (4EBP), and expression of postsynaptic density protein 95 (PSD95) and NMDAR2B in the IC on POD3 after nerve injury. (A,B)** p-mTOR increased in the NP group compared with the Sham group, but total mTOR levels were not significantly different on POD3. **(C,D)** p-P70S6K increased in the NP group compared with the Sham group, but total P70S6K levels were not different. **(E,F)** p-4EBP increased in the NP group compared with the Sham group, but total 4EBP levels were not different. **(G,H)** PSD95 and NMDAR2B levels increased significantly in the NP group compared with the Sham group. The intensity of the phospho-form band was normalized to that of the total form, and the total form bands were normalized to β-actin protein. Data are presented as means ± SEM. *P < 0.05, **P < 0.01 vs. Sham.

On the basis of the above results, we hypothesized that chronic pain derived from nerve injury alters mTOR signaling in the IC. To test this, we measured protein and phosphorylation levels of mTOR and its downstream targets, P70S6K and 4EBP, as well as PSD95 and NMDAR2B expressions in the IC at three and 7 days after nerve injury. The results indicate that on POD 3, p-mTOR was upregulated in the NP group (*n* = 5) compared with that in the sham group (*n* = 8; *P* < 0.05, unpaired* t* test), with no difference in the total amounts of mTOR (Figures 3A,B). Furthermore, phosphorylation levels of its downstream targets P70S6K and 4EBP were upregulated significantly in the NP group compared with those in shams (p-P70S6K: NP, *n* = 5; sham, *n* = 5, *P* < 0.05, unpaired* t* test; p-4EBP: NP, *n* = 4; sham, *n* = 4, *P* < 0.01, unpaired* t* test; Figures 3C,E). There were no changes in total P70S6K and 4EBP levels (Figures 3D,F, *P* > 0.05). Additionally, the expression of PSD95 in the NP group (*n* = 5) was higher than in the sham group (*n* = 5; Figure 3G, *P* < 0.05, unpaired* t* test). The level of NMDAR2B was increased in the NP group (*n* = 7) compared with the Sham group (*n* = 7; Figure 3H, *P* < 0.05, unpaired *t* test).

**Figure 4:**
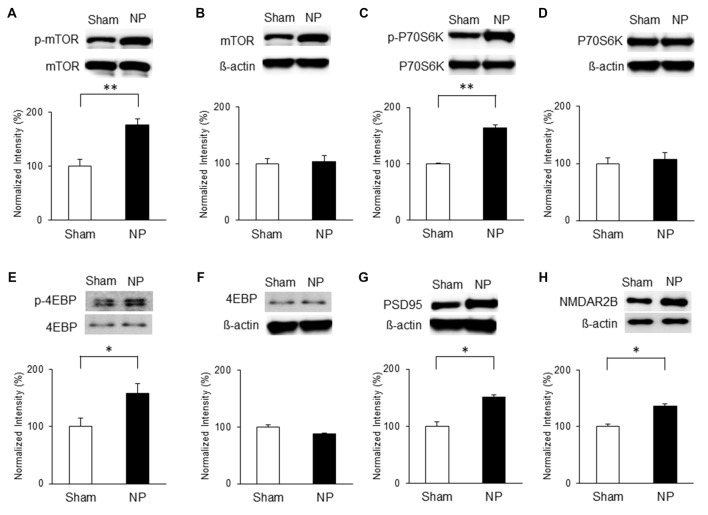
**Phosphorylation of mTOR, P70S6K and 4EBP and expressions of PSD95 and NMDAR2B in the IC on POD7 after nerve injury. (A,B)** p-mTOR increased in the NP group compared with the Sham group, but total mTOR levels were not significantly different on POD7. **(C,D)** p-P70S6K increased in the NP group compared with the Sham group, but total P70S6K levels were not different. **(E,F)** p-4EBP increased in the NP group compared with Sham group, but total 4EBP levels were not different. **(G,H)** PSD95 and NMDAR2B levels increased significantly in the NP group compared with the Sham group. The intensity of the phospho-form band was normalized to that of the total form, and the total form bands were normalized to β-actin protein. Data are presented as means ± SEM. *P < 0.05, **P < 0.01 vs. Sham.

Similarly, mTOR signaling activity was elevated in the NP groups compared with that in the shams on POD7. Levels of p-mTOR (Figure 4A) and p-P70S6K (Figure 4C) in the NP rats (*n* = 4) were higher than in the sham group (*n* = 4; *P* < 0.01, unpaired* t* tests). 4EBP activity measured by p-4EBP expression was also increased in the NP group (Figure 4E; *n* = 4 each, *P* < 0.05, unpaired* t* test). There were no differences in total levels of mTOR, P70S6K, and 4EBP (Figures 4B,D,F; *P*s > 0.05). The levels of PSD95 on POD7 were significantly higher in NP rats than in sham controls (Figure 4G; *n* = 4 each, *P* < 0.05, unpaired* t* test). Finally, the expression level of NMDAR2B increased significantly in the NP group (*n* = 6) compared with the Sham group (*n* = 6; Figure 4H, *P* < 0.05, unpaired* t* test). These results suggest that mTOR pathway in the IC strongly is related to PSD95 and NMDAR, and lead to neuropathic pain.

### Microinjection of Rapamycin in the IC Diminishes Mechanical Allodynia in Rats with Neuropathic Pain

**Figure 5:**
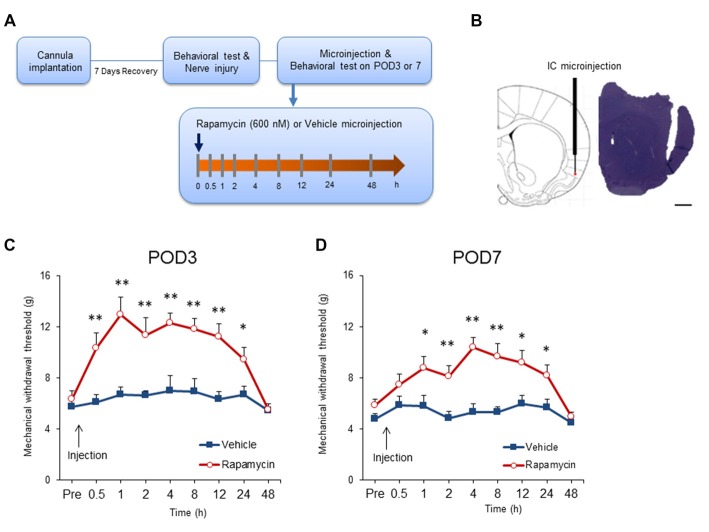
**Intracranial administration of rapamycin attenuates mechanical hypersensitivity. (A)** Experimental schematic highlighting the time points of cannula implantation, nerve injury, microinjection and behavioral testing. **(B)** Microinjection site into the IC (*left*) and a representative photograph of a coronal section (*right*). Scale bar, 1 mm. **(C)** Changes in paw withdrawal thresholds to mechanical stimulation after microinjection of rapamycin (Rapa group, Rapa) or vehicle (Vehicle group, Vehicle) on POD3. The arrow indicates the time point of microinjection. Significant differences between the rapamycin and vehicle groups were observed between 0.5 h and 24 h after microinjection. **(D)** Changes in paw withdrawal thresholds to mechanical stimulation after microinjection of rapamycin or vehicle on POD7. The arrow indicates the time point of microinjection. Similarly, the rapamycin and vehicle groups were significantly different between 1 h and 24 h after microinjection. Data are presented as means ± SEM. *P < 0.05, **P < 0.01 vs. Vehicle.

Previous studies demonstrated that intrathecal or systemic rapamycin administration reduces neuropathic pain (Asante et al., 2010; Melemedjian et al., 2013). To determine if this is due to effects in the IC, rats with neuropathic pain received microinjections of rapamycin (600 nM; *n* = 11–12) or the vehicle (*n* = 9–11) after mechanical allodynia testing on POD3 and POD7 (Figures 5A,B). Microinjection of rapamycin into the IC significantly affected withdrawal thresholds on POD3 (group, *F*_(1,20)_ = 18.171, *P* < 0.001; time, *F*_(8,160)_ = 10.8209, *P* < 0.001; group × time interaction, *F*_(8,160)_ = 5.191, *P* < 0.001; *n* = 11 each, two-way repeated measured ANOVA; Figure 5C). The analgesic effect of rapamycin in comparison with the vehicle persisted for up to 24 h after the microinjection as measured by mechanical allodynia (0.5 h, *P* < 0.01; 1 h, *P* < 0.01; 2 h, *P* < 0.01; 4 h, *P* < 0.01; 8 h, *P* < 0.01; 12 h, *P* < 0.01; 24 h, *P* < 0.05, unpaired *t* tests). However, there was no difference in the withdrawal thresholds between the rapamycin and vehicle groups 48 h after injection (respectively, *P* > 0.05, unpaired *t* test).

Similar results were observed when microinjections were performed on POD7. Figure 5D shows that microinjection of rapamycin on POD7 decreased mechanical allodynia significantly in comparison with rats receiving the vehicle (group, *F*_(1,19)_ = 14.546, *P* < 0.01; time, *F*_(8,152)_ = 6.510, *P* < 0.001; group × time interaction, *F*_(8,152)_ = 3.223, *P* < 0.01; rapamycin, *n* = 12; vehicle, *n* = 9, two-way repeated measured ANOVA). Mechanical thresholds in the rapamycin-treated group increased compared with the vehicle group from 1 h to 24 h after the injections (0.5 h, *P* > 0.05; 1 h, *P* < 0.05; 2 h, *P* < 0.01; 4 h, *P* < 0.001; 8 h, *P* < 0.01; 12 h, *P* < 0.05; 24 h, *P* < 0.05; 48 h, *P* > 0.05, unpaired *t* tests).

### mTOR Inhibition Reduces Pain-Related Activation of the IC of Nerve-Injured Animals

**Figure 6:**
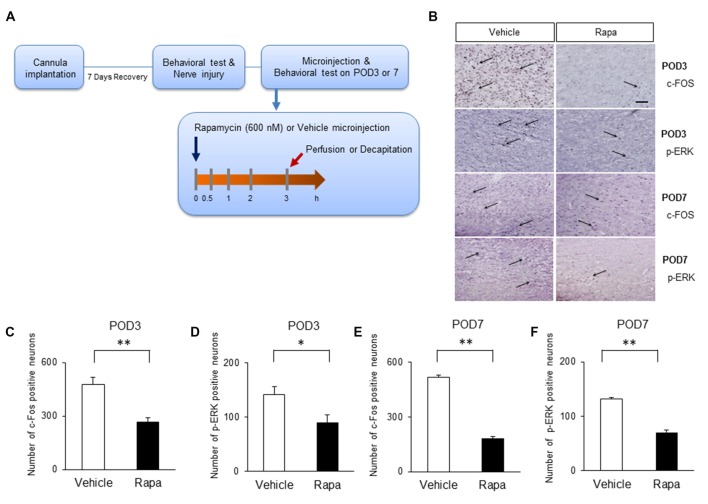
**Microinjection of rapamycin reduces the markers of nociceptive activation. (A)** Experimental schematic highlighting the time points of cannula implantation, nerve injury, microinjection and perfusion. **(B)** Microphotographs of c-FOS and p-ERK in the rostral IC. The arrows mean positive cells for c-FOS or p-ERK. On POD3, **(C)** the numbers of c-Fos-positive and **(D)** p-ERK-positive cells decreased significantly in the Rapa group compared with in the Vehicle group. On POD7, **(E)** the numbers of c-Fos-positive and **(F)** p-ERK-positive cells decreased significantly in the Rapa group compared with in the Vehicle group. Data are presented as means ± SEM. *P < 0.05, **P < 0.01 vs. Vehicle.

We assessed whether the inhibition of mTOR by rapamycin reduces the expression of markers of nociceptive activation in the IC 3 h after rapamycin/vehicle injections (Figure 6A). On POD3, rats receiving rapamycin injections had reduced number of cells positive for c-Fos (Figure 6B, the first row microphotographs and Figure 6C, the graph of c-Fos positive neurons, Rapa group (*n* = 6), Vehicle group (*n* = 6), *P* < 0.01, unpaired *t* test) and p-ERK (Figure 6B, the second row microphotographs and Figure 6D, the graph of p-ERK positive cells, Rapa group (*n* = 6), Vehicle group (*n* = 6), *P* < 0.05, unpaired *t* test) in the IC compared with in rats receiving the vehicle. Similarly on POD7, rats receiving rapamycin injections had reduced numbers of cells positive for c-Fos (Figure 6B, the third row microphotographs and Figure 6E, the graph of c-Fos positive cells, Rapa group (*n* = 4), Vehicle group (*n* = 4), *P* < 0.001, unpaired *t* test) and p-ERK (Figure 6B, the fourth row microphotographs and Figure 6F, the graph of p-ERK positive neurons, Rapa group (*n* = 4), Vehicle group (*n* = 4), *P* < 0.001, unpaired *t* test).

### Intracranial Administration of Rapamycin Blocks mTOR Signaling, PSD95 and NMDAR2B Expression in the IC of Nerve-Injured Animals

**Figure 7:**
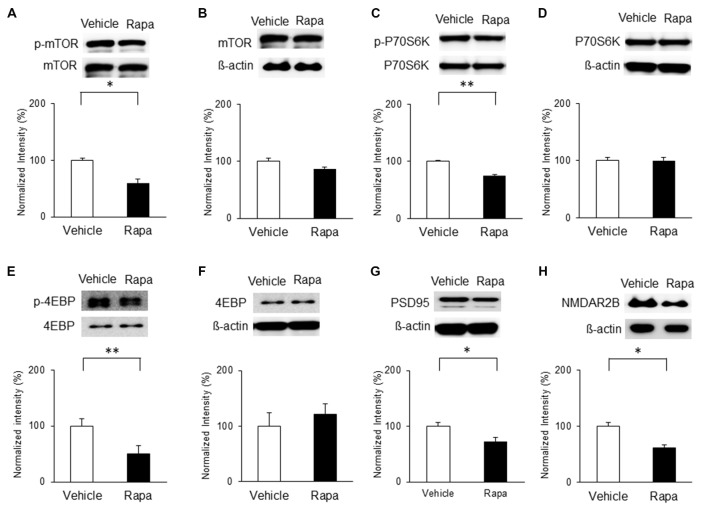
**Microinjection of rapamycin reversed the upregulation of the mTOR pathway and expressions of PSD95 and NMDAR2B on POD3 after nerve injury. (A,B)** p-mTOR levels decreased in the Rapa group compared with in the Vehicle group, but total mTOR levels were not significantly different. **(C,D)** p-P70S6K levels decreased in the Rapa group compared with in the Vehicle group, but total P70S6K levels were not different. **(E,F)** p-4EBP levels decreased in the Rapa group compared with in the Vehicle group, but total 4EBP levels were not different. **(G)** PSD95 and **(H)** NMDAR2B levels decreased in the Rapa group compared with in the Vehicle group. Data are presented as means ± SEM. *P < 0.05, **P < 0.01 vs. Vehicle.

We also assessed the levels of mTOR activation in the IC 3 h after rapamycin/vehicle injections; Figure 6A). On POD3, rats receiving rapamycin injections had lower expression of p-mTOR than vehicle-treated rats (Figure 7A; *n* = 5, *P* < 0.05, unpaired* t* test), as well as decreased phosphorylation of P70S6K and 4EBP (Figures 7C,E; *n* = 4 each, *P* < 0.01, unpaired* t* tests). However, the levels of mTOR, P70S6K and 4EBP were not significantly different (Figures 7B,D,F; *P* > 0.05). Additionally, the expressions of PSD95 and the NMDAR2B were decreased significantly with rapamycin compared with the vehicle (Figures 7G,H; *n* = 5 each, *P* < 0.05, unpaired *t* tests).

**Figure 8:**
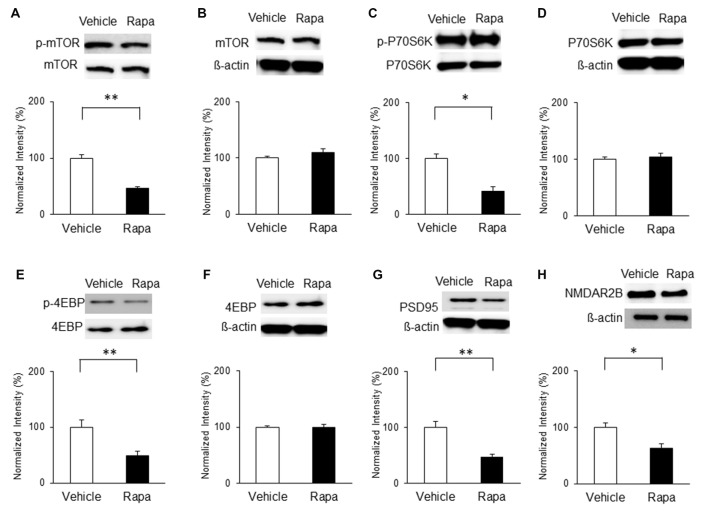
**Microinjection of rapamycin reversed the upregulation of the mTOR pathway and expressions of PSD95 and NMDAR2B on POD7 after nerve injury. (A,B)** p-mTOR levels decreased in the Rapa group compared with in the Vehicle group, but total mTOR levels were not significantly different. **(C,D)** p-P70S6K levels decreased in the Rapa group compared with in the Vehicle group, but total P70S6K levels were not different. **(E,F)** p-4EBP levels decreased in the Rapa group compared with in the Vehicle group, but total 4EBP levels were not different. **(G)** PSD95 and **(H)** NMDAR2B levels decreased significantly in the Rapa group compared with in the Vehicle group. The intensity of the phospho-form band was normalized to that of the total form, and the total form bands were normalized to β-actin protein. Data are presented as means ± SEM. *P < 0.05, **P < 0.01 vs. Vehicle.

On POD7, rats received microinjections of rapamycin or vehicle and mTOR activity and PSD95 expression were measured. The phosphorylation of mTOR was decreased significantly on POD7 in rats receiving rapamycin (Figure 8A; *n* = 4, *P* < 0.001, unpaired* t* test). Similarly, phosphorylation levels of P70S6K (Figure 8C; *n* = 4, *P* < 0.05, unpaired *t* test) and 4EBP (Figure 8E; *n* = 4, *P* < 0.01, unpaired *t* test) in the rapamycin-treated group were downregulated compared with in the vehicle-treated group. The expression levels of total mTOR, P70S6K and 4EBP were not significantly different (Figures 8B,D,F; *P* > 0.05). In addition, the expressions of PSD95 and the NMDAR2B in the rapamycin group were significantly decreased on POD7 compared with in the vehicle group (Figure 8G; *n* = 4, *P* < 0.01, Figure 8H; Rapa group (*n* = 5), Vehicle group (*n* = 5), *P* < 0.05, unpaired *t* tests).

### Administration of Rapamycin Reduced Excitability of the IC Induced by Peripheral Electrical Stimulation

**Figure 9:**
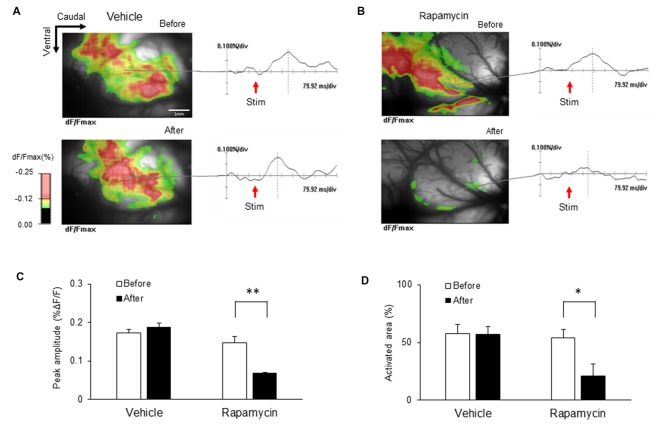
**Changes in optical signals before and after treatment with rapamycin and vehicle. (A)** Optical signals before and after vehicle treatment. **(B)** Optical signals before and after rapamycin treatment.** (C)** Peak amplitudes before and after vehicle and rapamycin treatments following peripheral electrical stimulation with 5.0 mA intensity. **(D)** Activated areas before and after vehicle and rapamycin treatments following peripheral electrical stimulation with 5.0 mA intensity. The peak amplitudes and activated areas induced by peripheral electrical stimulation after rapamycin treatment were reduced compared with those before rapamycin treatment. However, they are not significantly different in both before and after vehicle treatment. *P < 0.05, **P < 0.01.

The ICs of nerve-injured rats were imaged to elucidate the optical signal changes following electrical stimulation of the hind paw (5.0 mA) of the hind paw (Figure 9). Each image in response to stimulation before and after drug treatment is shown and color coded to indicate optical signals according to the changes in reflected light intensity (%ΔF/F). Figure 9A shows representative images taken before and after vehicle treatment. Figure 9B shows the corresponding images before and after rapamycin treatment. Wave forms were obtained from the points indicated in each image. The IC after the rapamycin treatment was rarely activated compared with that before treatment.

Peak amplitudes and the activated areas of the IC are represented in Figures 9C,D, respectively. Peak amplitudes induced by peripheral electrical stimulation after rapamycin treatment were reduced compared with those before rapamycin treatment (Figure 9C; *n* = 8, *P* < 0.01). The activated areas were also significantly reduced following peripheral electrical stimulation after rapamycin treatment compared with those before rapamycin treatment (Figure 9D; *n* = 8, *P* < 0.01). However, peak amplitudes and activated areas were not significantly different before and after vehicle treatment (Figures 9C,D).

## Discussion

Many studies have focused on the processes involved in neuropathic pain, and controlling neuropathic pain remains important (Ro and Chang, 2005). This study demonstrates that neuropathic pain after nerve injury is accompanied by considerable changes in the biochemistry and neuronal activity of the IC. More specifically, nerve injury increases the phosphorylation an activation of mTOR in the IC. To our knowledge, this is the first study to investigate the role of the mTOR signaling pathway and the potential effects of rapamycin in the IC using a neuropathic pain model.

Rapamycin has been used as an anticancer and immunosuppressive agent in clinical treatment (Populo et al., 2012; Tateda et al., 2017). Because the side effects from rapamycin are relatively low compared to with other drugs, it has also been widely used in chronic pain treatments (Lee et al., 2006; Alamo et al., 2009). Intrathecal, intraplantar and systemic injection of rapamycin alleviate mechanical hypersensitivity in formalin-induced pain models (Asante et al., 2009) as well as capsaicin-induced hyperalgesia (Géranton et al., 2009). Rapamycin may also reduce mechanical allodynia under inflammatory pain conditions (Obara et al., 2011).

Rapamycin is an inhibitor of mTOR, a molecule fundamental in cell growth and metabolism (Laplante and Sabatini, 2012). The activation of mTOR triggers phosphorylation of downstream effectors, such as P70S6K and 4EBP, to regulate mRNA translation and protein synthesis (Shih et al., 2012; Zhuang et al., 2016). Despite accumulating evidence supporting the role of mTOR signaling in pain-related memory processing (Zhang et al., 2013; Lutz et al., 2015), little is known about the molecular mechanisms of this pathway in the brain. In this study, we used behavioral, immunohistochemical, western blot and optical imaging analyses to demonstrate that mTOR signaling in the IC contributes to neuropathic pain and regulates mechanical hypersensitivity. Inhibition of this pathway by microinjections of rapamycin increased the pain threshold and reduced mechanical hypersensitivity on POD3 and POD7. Our findings are compatible with the results of previous data regarding mTOR (Asante et al., 2010; Norsted Gregory et al., 2010; Zhang et al., 2013). However, the analgesic effect did not persist beyond 24 h after microinjection, and further studies are needed to assess the duration of rapamycin’s efficacy.

The IC is involved in several sensory and cognitive processes, such as learning, memory and perception (Bermúdez-Rattoni, 2004; Craig, 2011), and connects with other regions to influence other higher-level functions, such as pain-perception and decision-making (Zhuo, 2016). While the IC plays an important role in pain processing (Alvarez et al., 2009; Coffeen et al., 2011; Qiu et al., 2014), the plasticity of this region in pain-related animal models remains unclear. It was shown that nociceptive stimuli activate neurons in the IC, as evidenced by increases in c-Fos (Gao and Ji, 2009), an immediate early marker of activation (Greenberg et al., 1986; Hunt et al., 1987; Coggeshall, 2005). A previous study indicated that the role of p-ERK was cell proliferation and differentiation (Widmann et al., 1999). Additionally, p-ERK has been implicated in synaptic plasticity related to memory and pain hypersensitivity (Ji and Woolf, 2001; Ji et al., 2003; Price and Inyang, 2015). We show that c-Fos and p-ERK levels are increased in the IC following nerve injury, suggesting that this brain region is closely related to the pain state. Importantly, these changes were attenuated by microinjections of rapamycin, indicating the mTOR signaling pathway as a mechanism for plastic changes regulating neuropathic pain.

Accordingly, we observed that nerve injury increased the expression of PSD95, an essential scaffold protein in the post synaptic density of excitatory synapses (Tao et al., 2003; Zhang et al., 2013). A previous study showed that the expression of PSD95 significantly increases when mTOR-dependent pathways are activated under stress conditions (Yang et al., 2008). Our results similarly demonstrate that neuropathic pain increases mTOR signaling and PSD95 expression. Moreover, levels of PSD95 in the IC were decreased by inhibition of mTOR with rapamycin. Interestingly, the knockdown of PSD95 in the spinal cord delays the progression of neuropathic pain (Tao et al., 2001, 2003). In our studies, mechanical allodynia as a result of peripheral nerve injury appears to be regulated by mTOR-dependent synthesis of PSD95 in the IC. Taken together, these data suggest that mTOR signaling maintains chronic pain conditions via regulation of synaptic proteins.

Interestingly, there is a report that mTOR activation requires activation of NMDA receptors in a bone cancer-induced pain model (Shih et al., 2012). In a neuropathic pain model, microinjection of an NMDA receptor antagonist into the IC significantly reduced pain behavior (Liu et al., 2013). Furthermore, LTP of synaptic responses in the IC is NMDA receptor dependent (Liu et al., 2013), and inhibition of protein synthesis or of the mTOR pathway can diminish LTP and memory (Melemedjian and Khoutorsky, 2015). These data suggest that LTP in the IC regulates neuropathic pain. Our data support this, as NMDA receptor expression in the IC was increased after peripheral nerve injury and attenuated by a microinjection of rapamycin.

As an additional means to demonstrate neuronal activation in the IC under neuropathic pain conditions, we performed optical imaging using a voltage-sensitive dye. This electrophysiological technique enables us to visualize the activity of large populations of neurons, which is observed as changes in membrane potentials. Because it has a high spatiotemporal resolution, several studies have reported the excitatory propagation patterns of pain-related brain areas (Kobayashi et al., 2010; An et al., 2012; Fehérvári et al., 2015). Our previous study (Han et al., 2016) showed that peripheral nerve injury increases the excitability and induces plastic changes in the IC. Here, we demonstrate that mTOR contributes to the chronic pain state, specifically in relation to synaptic plasticity. However, no studies have reported on the spatiotemporal pattern of the IC after mTOR inhibitor treatment in a neuropathic pain model. Therefore, we recorded optical signals induced by electrical stimulation of the hind paw to investigate the excitatory patterns in the IC with and without rapamycin treatment. The results of this study show an increase in excitability after nerve injury, consistent with the results of our previous study (Han et al., 2016). Furthermore, we show that this excitability is reduced after treatment with an mTOR inhibitor.

Even though mTOR signaling in the IC is involved in pain modulation, mTOR signaling pathway in other brain areas cannot be excluded to be involved in pain perception and modulation. In this regard, we tried to observe changes in the expressions of mTOR signaling in the secondary somatosensory cortex (S2) after administration of rapamycin. After the microinjection of rapamycin into the S2, the expression levels of p-mTOR and total mTOR were not changed compared with the expression levels prior to the injection (Supplementary Figure S1). This result indicates that the involvement of mTOR signaling in pain modulation may vary in different areas of the brain. Therefore, further studies may be necessary to elucidate the role of mTOR signaling in other brain areas that are likely to be involved in pain perception and modulation.

In conclusion, this study suggests that the mTOR pathway is a critical molecular signaling pathway regulating synaptic plasticity in the IC and mechanical hypersensitivity after peripheral nerve injury and neuropathic pain.

## Author Contributions

MK, JH and BHL designed the experiments. MK, JH, UJK, MC and SWU performed the experiments. MK, JH, SJB and S-KH analyzed the data. MK, JH and BHL wrote the article. MK and JH contributed equally to this work.

## Conflict of Interest Statement

The authors declare that the research was conducted in the absence of any commercial or financial relationships that could be construed as a potential conflict of interest.

## Supplementary Material

FIGURE S1 **Effects of microinjected rapamycin on mTOR signaling in the secondary somatosensory cortex (S2). (A,B)** Expressions of p-mTOR **(A)** and total mTOR **(B)** after rapamycin injection on POD3. **(C,D)** Expressions of p-mTOR **(C)** and total mTOR **(D)** after rapamycin injection on POD7. The expressions of p-mTOR and total mTOR were not changed after microinjection of rapamycin into the IC on POD3 and POD7. *P* > 0.05.
