# Supplementary figures and images for: Inhibition of Mammalian Target of Rapamycin (mTOR) Signaling in the Insular Cortex Alleviates Neuropathic Pain after Peripheral Nerve Injury
Source: Front Mol Neurosci. 2017 Mar 21;10:79. doi: 10.3389/fnmol.2017.00079 (PMC5359287; doi:10.3389/fnmol.2017.00079)

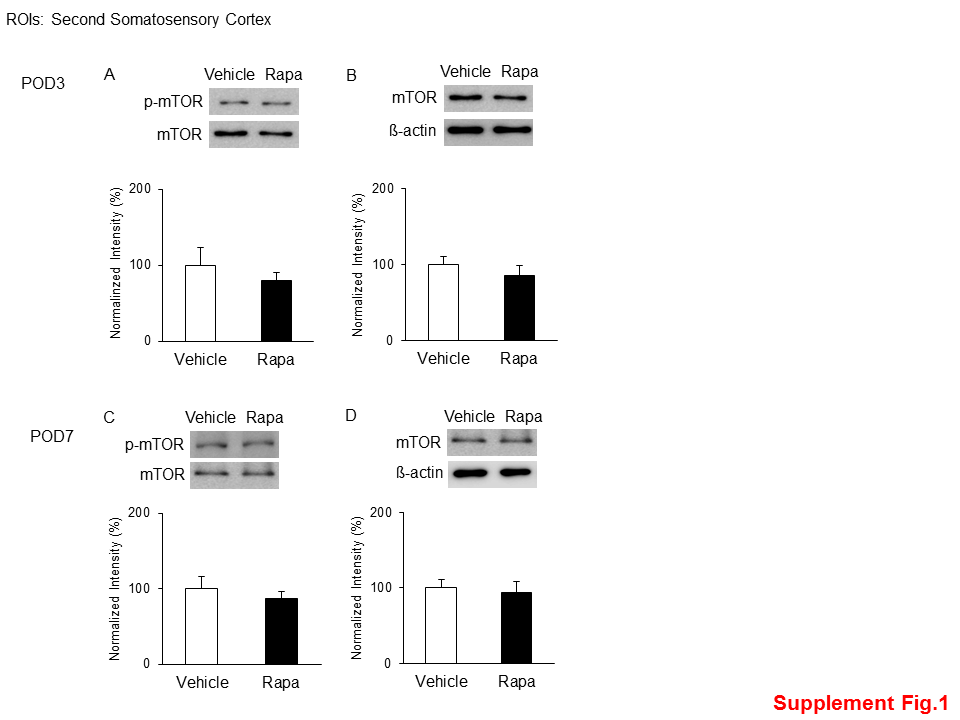

Supplement: FIGURE S1 — Effects of microinjected rapamycin on mTOR signaling in the secondary somatosensory cortex (S2). (A,B) Expressions of p-mTOR (A) and total mTOR (B) after rapamycin injection on POD3. (C,D) Expressions of p-mTOR (C) and total mTOR (D) after rapamycin injection on POD7. The expressions of p-mTOR and total mTOR were not changed after microinjection of rapamycin into the IC on POD3 and POD7. P > 0.05. [file Image_1.tif]
